# Looking at sound: optoacoustics with all-optical ultrasound detection

**DOI:** 10.1038/s41377-018-0036-7

**Published:** 2018-08-15

**Authors:** Georg Wissmeyer, Miguel A. Pleitez, Amir Rosenthal, Vasilis Ntziachristos

**Affiliations:** 1 Institute of Biological and Medical Imaging, Helmholtz Zentrum München, Neuherberg, Germany; 2 Chair of Biological Imaging, Technische Universität München, Munich, Germany; 3 Andrew and Erna Viterbi Faculty of Electrical Engineering, Technion - Israel Institute of Technology, Haifa, Israel

## Abstract

Originally developed for diagnostic ultrasound imaging, piezoelectric transducers are the most widespread technology employed in optoacoustic (photoacoustic) signal detection. However, the detection requirements of optoacoustic sensing and imaging differ from those of conventional ultrasonography and lead to specifications not sufficiently addressed by piezoelectric detectors. Consequently, interest has shifted to utilizing entirely optical methods for measuring optoacoustic waves. All-optical sound detectors yield a higher signal-to-noise ratio per unit area than piezoelectric detectors and feature wide detection bandwidths that may be more appropriate for optoacoustic applications, enabling several biomedical or industrial applications. Additionally, optical sensing of sound is less sensitive to electromagnetic noise, making it appropriate for a greater spectrum of environments. In this review, we categorize different methods of optical ultrasound detection and discuss key technology trends geared towards the development of all-optical optoacoustic systems. We also review application areas that are enabled by all-optical sound detectors, including interventional imaging, non-contact measurements, magnetoacoustics, and non-destructive testing.

## Optical sensors: Ultra-sharp views of ultrasound

Optoacoustic systems that use laser-induced ultrasound waves to reveal the 3D interiors of biological and man-made materials can find wider applications with optical sensors. Georg Wissmeyer from the Technische Universität München in Germany and colleagues review how high-bandwidth optical components are replacing conventional piezoelectric and capacitive sound detectors. One approach, which measures the phase of a probe beam as it reacts to ultrasound-driven changes in refractive index, can capture the entire acoustic field moving through a medium in a single snapshot. Another strategy employs micro-resonators to miniaturize interferometric sound sensors onto the tips of optical fibers for challenging intravascular imaging, such as during stent implantation. Because all-optical sensors are relatively insensitive to electromagnetic noise, they can be deployed in industrial environments for non-destructive testing of material defects.

## Introduction

Optoacoustic imaging defines new challenges for ultrasound detection compared to ultrasonography^1–5^. While ultrasound image formation operates over a relatively narrow frequency band (typically 50% of the central frequency), optoacoustic signal generation based on ultra-short laser pulses is broadband and can span frequencies from sub-MHz to hundreds of MHz. Optoacoustic signals collected in vivo can be up to three orders of magnitude weaker than the signals detected in medical ultrasound imaging because contrary to ultrasonography, optoacoustic signal generation occurs within the interrogated medium and is limited by the maximum light dose legally permissible for tissue illumination. In addition, optoacoustic imaging utilizes tomographic principles that generally require collection of data over wide acceptance angles (projections), which improves image quality and resolution as well as minimizes image artifacts. Four key performance parameters of ultrasound detectors are given below.

### Bandwidth and central frequency

These two parameters are critical for the resolution and size range of structures that can be detected in optoacoustic imaging. To detect optoacoustic signals from absorbers ranging widely in size down to the micron scale, sensors must have central frequencies as well as bandwidths on the order of hundreds of MHz^6^. For example, optoacoustic tomography of absorbers between 10 and 300 µm in size requires a detector with frequency responses from a few MHz to >150 MHz^7^. The desired frequency response depends on the intended application, because ultrasound waves propagating in tissue undergo frequency-dependent attenuation on the order of ~0.5 dB per MHz per cm^8^. Therefore, deeper imaging involves narrower bandwidths and lower frequencies than more superficial imaging. For example, a 5-MHz acoustic wave propagating through a depth of 10 cm will undergo attenuation of ~25 dB. A 50-MHz wave will undergo the same attenuation through a depth of 1 cm. Therefore, higher frequencies achieve higher imaging resolution, albeit at reduced penetration depths.

### Sensitivity

Detector sensitivity is commonly defined as the minimum detectable signal pressure amplitude. For acoustic sensors, the sensitivity is determined in relation to the noise level of the detector expressed in pressure units and is referred to as noise-equivalent pressure (NEP). In ultrasonography, a typical acoustic pulse reflected from an organ/tissue interface has a pressure magnitude at the sensor in the kPa range^8^. Although tissue illumination at the maximum laser fluence permitted by ANSI safety standards^9^, i.e., 100 mJ/cm² in the near-infrared (NIR), may generate optoacoustic signals from the tissue surface similar to those used in ultrasonography, accurate sensing of signals collected from deeper in tissues requires detection sensitivity in the Pa or sub-Pa range^10^.

### Size

As a critical parameter of ultrasound transducers, detector size is a major driver in the development of optical detectors. Miniaturization of piezoelectric transducers is fundamentally limited by two key considerations. First, the sensitivity achieved usually drops when reducing the active piezoelectric transducer area. Second, the casing of the piezoelectric element, preamplifiers and electrical isolation measures also affect the minimum size of the piezoelectric transducer. In contrast, optical detectors such as those based on interferometric resonators can be miniaturized without a similar dependence on active detection area, i.e., no loss of sensitivity with area. For example, piezoelectric elements designed for intravascular ultrasonography have diameters of ~1 mm and offer NEP of 1.8 kPa and bandwidth of 16 MHz. Correspondingly, optical-fiber-based transducers can offer 100 Pa NEP and 77 MHz bandwidth with a sensing area of only 0.13 × 0.27 mm^11,12^. Miniaturization of ultrasound transducers is particularly important in minimally invasive measurements such as for medical endoscopy or for inspection of small hollow structures and lumen in non-destructive testing. Small-area detectors are preferred in tomographic applications as well, whereby the spatial resolution depends inversely on detector size.

### Detector aperture

This factor, often also related to as acceptance angle, is another critical parameter in many optoacoustic applications. It refers to the range of angles over which a detector can detect signals, and it is typically defined as the angle formed by (a) the axis perpendicular to the detector area and (b) the axis on which the signal from an ultrasound source is detected with 6 dB attenuation with respect to the signal detected at angle zero. In optoacoustic tomography, a large aperture and well-characterized angle-dependent frequency response are essential for accurate image reconstruction and quantitative measurements^13^. For example, the image performance and axial resolution achieved by raster-scan optoacoustic mesoscopy operating in epi-illumination (reflection) mode depends on the aperture employed^7^. Piezoelectric elements are generally directional, typically offering acceptance angles below ± 20°^14^ and may require acoustic lenses to increase their acceptance angles^15^. In contrast, many optical detectors have inherently higher acceptance angles^16^.

Since its emergence in the late 1970s, optoacoustic imaging has relied primarily on detectors based on piezoelectric technology developed for ultrasonography. Piezoelectric transducers can not only detect but also generate and guide ultrasound beams, which has led to the development of portable medical ultrasound systems and resulted in cost-efficient designs of one- or two-dimensional transducer arrays. However, piezoelectric transducers are not always well suited to optoacoustic detection. They have relatively limited bandwidths and acceptance angles that capture only a part of the optoacoustic signals generated in tissues, which results in loss of image quality. The central frequency of a piezoelectric element is typically determined by its thickness, and higher bandwidths can be achieved only at small acceptance angles^17^. Moreover, common piezoelectric transducers are opaque and block optical paths required in optoacoustics, although transparent piezoelectric elements based on tin-oxide-coated polyvinylidene difluoride (PVDF) or indium-tin-oxide-coated lithium niobate have been considered^18^.

Similarly, the sensitivity of piezoelectric transducers generally depends on the size of the active transducer area, which limits miniaturization. This loss of sensitivity is mitigated in emerging micromachined sensors, including piezoelectric micromachined ultrasound transducers (PMUTs)^19^ and capacitive micromachined ultrasonic transducers (CMUTs). PMUTs utilize diaphragm-like geometries that enable the implementation of sensitive miniaturized thin-film transducers typically formed on silicon substrates. CMUTs utilize capacitor cells on silicon substrates using semiconductor fabrication techniques^20^, offering comparably sensitive detection from sub-100 micron detection areas. Both technologies offer high-density array designs in one or two dimensions using miniaturized detectors and seamless implementation with electronic circuits^14,21^ (Table 1). However, the detection bandwidths currently achieved are limited to a few tens of MHz, which may limit optoacoustic applications, in particular optoacoustic microscopy and mesoscopy^3^. In some ways, CMUT technology has advanced farther than that of PMUTs, yet both technologies likely require further research to improve their performances, sensitivities, and bandwidths^19^.

**Table 1:** Comparison of optical, piezoelectric, and micromachined ultrasound transducers

Refractometric transducer	Material	Read-out element	Bandwidth (MHz)	Sensing element size (mm)	NEP (mPa/Hz^1/2^)	NEP × area (mPa mm^2^/Hz^1/2^)
Intensity-sensitive^24^	Silica	Photodiode	100	Prism (15)	100	22 × 10³
Beam deflectometry^30^	Coupling medium	QPD	17	Needle beam (0.09)	2.76	N.A.
Phase-sensitive^35^	Coupling medium	CCD/CMOS	110	Schlieren beam (10)	486	N.A.
**Interferometric transducer**	**Material**	**Dimensions (H** **×** **W** **×** **L, µm)**	**Q-factor**	**Conversion efficiency (M/Pa)** ^**−1**^	**NEP (mPa/Hz** ^**1/2**^ **)**	**NEP** **×** **area (mPa mm**^**2**^**/Hz**^**1/2**^**)**
Micro-ring^70,137^	Polystyrene	1.4 × 20 × 20 −1.4 × 100 × 100	1.4 × 10^5^	130 × 10^−6^	5.61	1.8 × 10^−3^
Fabry-Pérot^62–65^	PET/Parylene C	38 × 90 × 90	2.8 × 10^3^	90 × 10^−6^	78	0.63
	SU8	10 × 15 × 15	300	N.R.	200	4.5 × 10^−2^
	Fluid with low optical absorption	60 × 60 × 2 × 10^3^	N.R.	N.R.	0.45	5.4 × 10^−2^
π-BG^11,79,81^	Silica	10 × 10 × 270	1.2 × 10^6^	3.8 × 10^−6^	25	6.75 × 10^−2^
	Silicon	0.2 × 0.5 × 30	1.2 × 10^5^	N.R.	N.R.	N.R.
**Piezoelectric transducer**	**Model**	**Detector type**	***f*** **(MHz)**	**Area (mm** ^2^ **)**	**NEP (mPa/Hz** ^**1/2**^ **)**	**NEP** **×** **area (mPA·mm²/Hz**^**1/2**^**)**
Olympus NDT panametrics	V214-BB-RM	Spherically focused piezoceramic	50	30	0.2	6
Precision acoustics	Needle (1 mm)	PVdF needle hydrophone	12	1	14.4	14.4
Boston Scientific^12^	Atlantis PV	Intravascular ultrasound probe	15	0.8	450	360
**Micromachined transducer**	**Material**	**Detector type**	***f*** **(MHz)**	**Area (mm** ^2^ **)**	**NEP (mPa/Hz** ^**1/2**^ **)**	**NEP** **×** **area (mPA·mm²/Hz**^**1/2**^**)**
CMUT^20,21^	Silicon	Array of capacitor cells	5	0.06	1.8	0.11

*NEP* noise equivalent pressure, *QPD* quadrant photodiode, *CCD* charge-coupled device, *CMOS* complementary metal-oxide-semiconductor, *PET* polyethylene terephthalate, *SU8* epoxy-based photoresist, *π-BG* pi-phase-shifted Bragg grating, *CMUT* capacitative micromachined ultrasonic transducer, *N.A.* not applicable, *N.R.* not reported

Optical techniques are increasingly considered a promising alternative to sound detection in optoacoustic systems, with the potential to address limitations of piezoelectric and capacitive technologies. Interestingly, optical sound detection leads to optoacoustic systems in which sound is both generated and detected exclusively by optical components and may offer higher sensitivity and broader detection bandwidths with smaller form factors than piezoelectric transducers, PMUTs or CMUTs. In this review, we examine progress with optical technology for sound detection employed in all-optical optoacoustic systems. Some related technologies have recently been reviewed for an expert audience^22^; however, our goal in the present review is to bring these advances to a broader audience and present them in a comprehensive context with a broader range of reported applications. First, we classify different techniques according to their principles of operation, explain their technical specifications and discuss their major physical and operational characteristics, including their advantages and limitations. Then, we profile the unique biomedical sensing and imaging applications made possible by advances in optoacoustic applications. Finally, we examine how these advances may lead to new applications in non-biomedical applications, such as in the emerging field of magnetoacoustics as well as in the field of non-destructive testing.

## Optical sensing of ultrasound

**Fig. 1:**
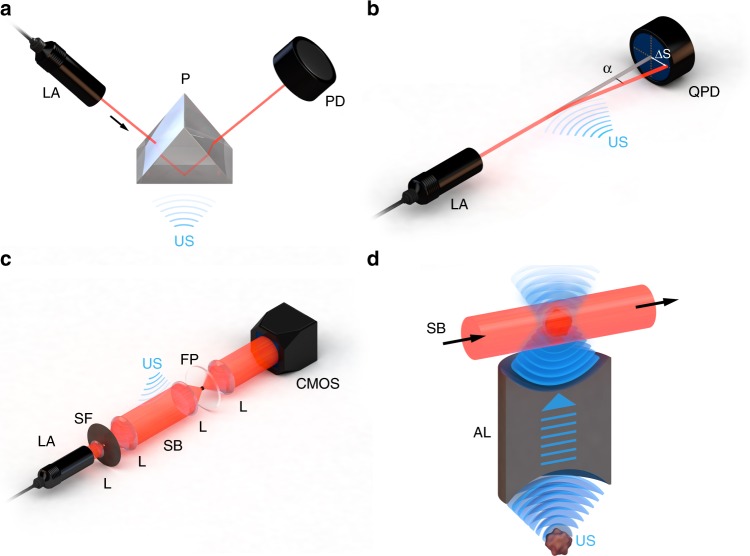
Refractometric ultrasound detectors. **a** Intensity-sensitive detection of refractive index. **b** Single-beam deflectometry. **c** Phase-sensitive ultrasound detection with a Schlieren beam. **d** Phase-sensitive ultrasound detection with a decoupled optoacoustic source. AL acoustic lens, CMOS CMOS camera, FP Fourier plane, L lens, LA laser, P prism, PD photodiode, QPD quadrant photodiode, SB Schlieren beam, SF spatial filter, US ultrasound

The two optical methods commonly applied for detecting ultrasound waves are refractometry and interferometry. Refractometry-based detection uses the photoelastic principle, which states that acoustic waves interacting with a medium induce mechanical stress in that medium, causing the refractive index (RI) to change proportionally with the mechanical pressure^23^. The method uses a laser beam (called an interrogating or probe beam) to measure changes in the RI of a single medium or at the interface between two adjacent media in response to propagating acoustic waves (Fig. 1). Changes in the intensity, deflection angle or phase of the probe beam are recorded at an optical detector, providing information about the ultrasound signals interrogated.

**Fig. 2:**
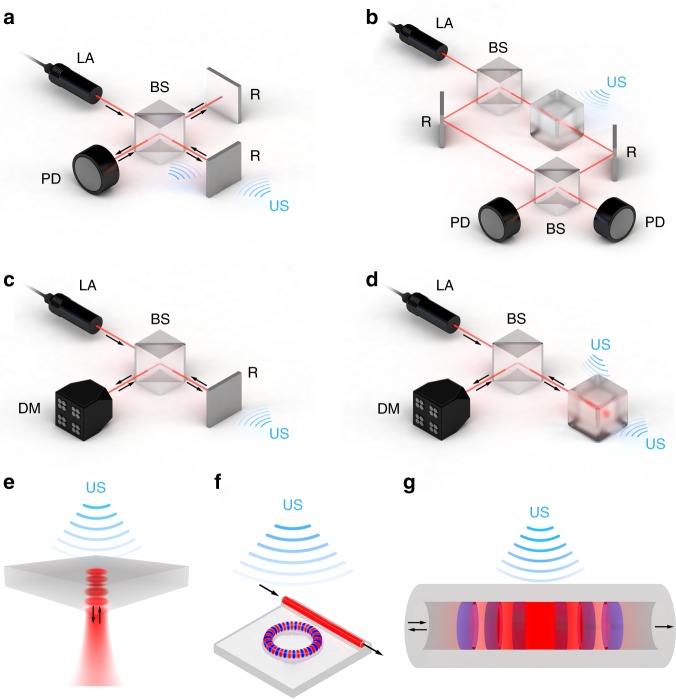
Interferometric ultrasound detectors. **a**–**d** Sensing mechanisms: **a** phase detection in a Michelson interferometer, **b** phase detection in a Mach–Zehnder interferometer, **c** Doppler-based sensing, and **d** resonator-based sensing. The ultrasound wave is applied at **a**, **b** the beam path, **a**, **c** the reflector, or **d** the resonator. Detection is performed by either **a**, **b** photodetectors or **c**, **d** demodulators as described in section “Interferometric methods”. **e**–**g** Common resonator geometries demonstrated for ultrasound sensing: **e** planar Fabry-Pérot, **f** micro-ring, and **g** π-phase shifted fiber Bragg grating. The optical beam and material of the resonators are red and gray, respectively, whereas the semi-periodic refractive index modulation that makes up the π-FBG is blue. The figure also depicts the standing-wave effect that exists in the Fabry-Pérot and micro-ring resonators, as well as the light localization around the π-phase shift of the π-FBG. BS beam splitter, D detector, DM demodulator, LA laser, R reflector, US ultrasound

Interferometric methods detect changes in optical interference patterns induced by ultrasound. Ultrasound waves alter the interference condition by interacting directly with an optical beam, by causing vibrations of a reflector or by altering the resonance frequency of a resonator (Fig. 2). Perturbations in the interference pattern may be triggered by changes in the mean free path, the optical phase or the optical wavelength, depending on the interferometric configuration employed. The resulting changes in intensity or frequency at the interferometer output are detected by a photodiode or a wavelength meter and reveal information about the ultrasound signals.

In the following, we discuss the principles of operation of refractometric and interferometric methods in all-optical optoacoustic detection and illustrate their major advantages and disadvantages for biomedical applications. Table 1 summarizes key performance parameters of these methods and contrasts them with piezoelectric detection of ultrasound.

### Refractometric methods

#### Intensity-sensitive methods

The intensity of an optical beam incident on an interface between two media with different RI (e.g., a water-glass interface using a prism) fluctuates when ultrasound waves of varying intensity pass through the interface. Then, ultrasound measurement is achieved by recording the fluctuations in optical intensity using an optical detector such as a photodiode (Fig. 1a).

Different variations on the basic design in Fig. 1a have been reported. One design splits the probe beam into two polarized components and measures ultrasound-induced changes in intensity in each reflected component separately. This allows the calculation of the reflectance ratio of the two components, which reduces noise in the probe beam and thereby increases the signal-to-noise ratio (SNR)^24^. Another design creates a surface plasmon resonance at the interface so that ultrasound waves affect the probe beam reflectivity by altering the plasmon resonance condition. In this approach, the bottom of the prism is coated with a metal–dielectric interface and a polarized probe beam is used to induce surface plasmons at the interface. When the resonance condition for the creation of plasmons at the dielectric interface is satisfied, pressure-induced changes at the interface modulate the light-plasmon coupling and thus modulate the intensity of the reflected probe beam^25,26^.

Intensity-sensitive methods can detect ultrasound over a wide dynamic range of pressures, from single-digit kPa up to 180 MPa^24,27,28^, making them attractive for applications where high ultrasound pressures are recorded, e.g., sound shockwaves generated during the optoacoustic monitoring of intravascular laser ablation^28^. On the other hand, intensity-sensitive methods show limited potential for optoacoustic imaging. In optoacoustic microscopy, the optical beam path needs to be guided through a prism (Fig. 1a), making it necessary to use objectives with relatively low numerical apertures and longer working distances^24^. Moreover, these methods have demonstrated poor sensitivity for typical optoacoustic signal pressures that are usually on the order of 100 Pa or less. The poor sensitivity makes the technique inadequate for demanding optoacoustic microscopy and flow measurement applications. Nevertheless, an optoacoustic microscope has been designed in which the ultrasound signal is detected extremely close to the area of excitation, before the acoustic waves have propagated into the surrounding tissue, i.e., in areas where pressures can be on the order of MPa^29^.

#### Deflection-based methods

Instead of detecting ultrasound based on changes in the intensity of a probe beam, which requires an interface between two media of different RI, ultrasound can additionally be detected based on the deflection of the probe beam crossing the acoustic field (Fig. 1b)^30,31^. The acoustic waves alter the RI of the medium and interact with the electric field of the probe laser beam, deflecting it in proportion to the pressure gradient of the acoustic wave. This deflection is detected using a position-sensitive detector such as a quadrant photodiode. The frequency bandwidth that can be detected is determined not only by the photodiode rise time, as in intensity-based methods, but also by the diameter of the probe beam, with smaller diameters able to detect wider bandwidths. Dividing the speed of sound in water by the beam diameter for a probe beam with a diameter of 90 µm, for example, gives a theoretical bandwidth of 17 MHz^30^. Deflection-based methods involve an extremely small active sensing area, which allows the use of objectives with high numerical apertures such as for optoacoustic microscopy.

The strong potential of these methods for optoacoustic microscopy is evidenced by the success of acousto-optic beam deflectometry (AOD). Although AOD is already well established as a method for non-destructive testing of materials, reported sensitivities of 12 Pa make the AOD technology comparable to piezoelectric transducers and potentially suitable for optoacoustic applications^30^. However, in AOD setups, the laser beam must be narrowly focused and guided through the medium close to the acoustic source. This requirement may limit implementation in tightly spaced optoacoustic microscopy and tomography setups that utilize optical guiding systems for sample illumination, further reducing the space available for an interrogation system. In addition, current tomographic image reconstruction algorithms do not take into account the interaction of the probe beam with multiple acoustic sources, which means AOD cannot yet be implemented in optoacoustic tomography. Instead, AOD may more quickly find application in optical-resolution optoacoustic microscopy, where image resolution is determined by the optical resolution of the system^30,31^.

#### Phase-sensitive methods

Another all-optical approach for detecting ultrasound involves measuring ultrasound-induced shifts in the phase of a collimated probe beam^32^. The phase shift *φ* and acoustic field *p* are related by the equation

1 $$\phi = \frac{{2{\boldsymbol{\pi }}}}{{\boldsymbol{\lambda }}}\frac{{{{{\rm{d}}n}}}}{{{{{\rm{d}}p}}}}{\int} {{\boldsymbol{p}} \cdot {{{\rm{d}}z}}}$$

where d*n/*d*p* is the elasto-optic coefficient of the medium in which the beam and acoustic field interact, and *λ* is the wavelength of the probe beam. In this approach, a highly collimated light beam is passed through an acoustic field. Due to the change in RI produced by the acoustic field in its propagation medium, some photons in the beam are scattered or deflected from the original path (Eq. 1). This perturbed probe beam is then tightly focused through a spatial filter in the Fourier plane (Fig. 1c) to selectively remove the out-of-phase photons for negative contrast of the acoustic field or the in-phase photons for positive contrast. After the spatial filtering, the beam is re-collimated by a lens and detected by a CCD or CMOS camera. In this way, the camera reproduces an intensity map of the acoustic field^33^ that can be converted into two- or three-dimensional images of the source of the ultrasound signals by standard tomographic reconstruction techniques.

Optical phase-sensitive ultrasound detection methods are already widely used in characterizing ultrasound transducers and studying the effects of acoustic shock waves on aircraft, where the acoustic field intensity is hundreds of kPa. Phase-sensitive systems can also detect ultrasound with an NEP as low as 5.1 kPa^34^ and a bandwidth as wide as 110 MHz^35^. With this sensitivity and bandwidth, phase-sensitive ultrasound detection can be implemented in optoacoustic tomography using various experimental approaches, such as Schlieren photography, phase contrast imaging, and shadowgraphy^33^. These optical approaches, which differ primarily in the filtering method applied in the Fourier plane, have been combined with light sheet excitation for optical sectioning^36^. Here, a two-dimensional ultrasound projection through depth (known as a *B-scan*) is imaged during each laser shot, and a three-dimensional image is obtained by moving the sample along the axis perpendicularly to the light sheet illumination.

Real-time three-dimensional optoacoustic tomography without the need for computational reconstruction has been achieved using an acoustic lens and all-optical phase-sensitive ultrasound detection^37^. In this method, the volumetric optoacoustic field is collected at one side of the acoustic lens and re-focused at the other side into a water tank, where the pressure field is imaged by the phase-sensitive system (Fig. 1d). In this way, the acoustic field is decoupled from the sample, allowing easy optoacoustic detection without the interferences that would arise if the sample were positioned directly in the Schlieren beam^38–41^. These interferences arise due to optical absorption as well as scattering of the Schlieren beam in the sample and are the key challenges of phase-sensitive methods. While acoustic lenses can address this issue, acoustic losses due to attenuation and inadequate lens materials need to be reduced.

Contrary to single-element transducers, these phase-sensitive optical methods for ultrasound detection can capture the entire acoustic field in a single snapshot in which the acoustic signal is spatially encoded. Here, the acoustic bandwidth is determined by the optical resolution of the system rather than by the transducer bandwidth as is currently the case in optoacoustic tomography^7^.

### Interferometric methods

Methods of optical interferometry for sound detection require a sensing system and a read-out (interrogation) system. In the sensing system, physical characteristics of ultrasound waves (e.g., displacement, pressure) alter physical characteristics of light (e.g., optical path length, phase, wavelength) that can be measured using optical interferometry. In the read-out system, these altered characteristics are detected and converted into voltage signals. Therefore, the NEP of an interferometric ultrasound detector is determined by the efficiency with which acoustic perturbations are converted into changes in light characteristics in the sensing system, as well as by the sensitivity of the read-out system for detecting those changes. Below, the two systems are discussed separately for simplicity, although certain sensing systems are usually used with certain read-out systems.

#### Sensing systems

The earliest interferometers, Michelson interferometers^42,43^ (MI) and Mach–Zehnder interferometers^44,45^ (MZI), were first used to measure vibrational displacement in the 1960s. In the 1970s, optical resonators^46,47^ and optical fibers^48–50^ emerged as more sensitive systems for detecting acoustic waves, opening the door to miniaturized portable devices. In the 1980s and 1990s, interferometers highly efficient at collecting scattered light^51–53^ allowed non-contact detection of ultrasound from rough surfaces. Since then, several non-contact approaches, among them Doppler interferometry (DI), have given rise to an entire field of laser ultrasonics^54^. Non-contact approaches avoid the need for the sensing element to come into physical contact with the sample, making an acoustic coupling medium unnecessary.

In MIs and MZIs, a laser beam is split into two optical paths, one of which is perturbed by the ultrasound wave and the other serves as a reference (Fig. 2a, b). The two beams are combined at the interferometer output and their interference is measured; ultrasound-induced changes in the optical path cause proportional changes in the intensity of the recombined beam. In MIs, ultrasound may interact with the beam itself or with a reflector that the beam strikes (Fig. 2a); in MZIs, ultrasound interacts with one of the beam arms (Fig. 2b). In MIs and MZIs, an acoustic coupling medium must be used when ultrasound interacts with the beam path, but not when it interacts with a reflector, which can be the sample itself^55–57^.

Two-beam interferometers are usually implemented in fiber-based^45,58,59^ or free-beam^44,60^ configurations. Fiber-based MZIs can achieve sensitivities down to 180 Pa^58^. While such sensitivity is encouraging for optoacoustic imaging, this approach has so far been validated only for ultrasound signals with a bandwidth of 5 MHz, which is insufficient, especially for optoacoustic tomography. In another approach, optoacoustic tomography involving a free-beam MZI has shown sensitivity of 100 Pa and bandwidth of 17 MHz^44^. These results were obtained under laboratory conditions of minimal vibration and electromagnetic noise, so further work is needed to establish the robustness of this approach.

In contrast to two-beam interferometers, Doppler interferometers sense ultrasound by detecting Doppler shifts in light frequency (Fig. 2c)^53,61^. In DI, the ultrasound waves interact with a reflector similar to some MI setups, but the reflected probe beam does not interfere with a reference beam; instead, Doppler shifts in the probe beam induced by pressure-induced oscillations of the reflector are recorded by a wavelength demodulator. DI offer advantages over MIs, particularly when the reflector is a rough surface. The rough surface creates a distorted wavefront that interferes with itself, providing greater interferometric contrast than when a distorted wavefront combines with the nearly planar wavefront of the reference beam as in MIs. DI shows great potential for non-contact optoacoustic imaging, but the lack of suitable read-out systems poses a problem (see next section).

A promising alternative to two-beam interferometry and DI is ultrasound sensing based on optical resonators. These resonators confine the probe beam to a small volume, prolonging the interaction between the beam and the acoustic wave, thereby increasing the sensitivity of the beam to acoustic perturbations. An optical resonator can detect ultrasound waves travelling either perpendicularly or parallel to the probe beam, depending on the geometry (Fig. 2d). The use of micron-scale resonators enables miniaturization of the entire sensor system, because the acoustic perturbation affects only the light inside the resonator. The optical resonator geometries most frequently used in optoacoustic imaging are classic Fabry-Pérot interferometers^62–69^ (Fig. 2e**)**, micro-ring resonators^16,70–72^ (MRRs) and π-phase-shifted fiber Bragg gratings^11,12,73–75^ (π-FBGs).

Fabry-Pérot interferometers have been demonstrated with sensitivities on the order of 50 Pa and bandwidths of up to 40 MHz^62^. The resonators in this approach trap the probe beam between two opposing flat mirrors or reflecting surfaces. This simple resonator geometry serves as the basis for diverse sensor designs. A particularly successful design is slab geometry based on thin transparent foils^62,76^ in which both sides of the foil are coated with a reflecting material. A focused probe beam interrogates this resonator at specific positions; scanning the probe beam over the foil effectively mimics a dense array of ultrasound transducers. The density of this array can be even higher than that of piezoelectric transducers and does not require mechanical scanning, which is particularly advantageous in optoacoustic tomography. The simplistic resonator design allows the manufacture of enhanced resonators on the tip of optical fibers, e.g., where the upper reflector is plano-concave, which strengthens light confinement and therefore improves the Q-factor and sensitivity^77^. Nevertheless, these resonators cannot achieve total light confinement, and the inevitable losses of light may make them unsuitable for applications demanding higher detector sensitivity, such as optoacoustic microscopy.

MRR (Fig. 2f) sensitivities down to 6.8 Pa and bandwidths of 140 MHz have been achieved^16^ in optoacoustic microscopy. The MRRs in that study were fabricated on a glass microscope slide, meaning that the detector was fully transparent and therefore ideal for microscopy in reflection mode. The small footprint of MRRs and their symmetrical, disc-shaped geometry with diameters of ~100 µm make them attractive for optoacoustic tomography^78^. On the other hand, MRRs typically provide a broad detection bandwidth only at narrow opening angles^16^, which is not ideal for tomography.

Sensing systems based on π-FBGs (Fig. 2g) have been shown to work well as part of endoscopic and optoacoustic microscopy frameworks, providing sensitivities of 100 Pa and bandwidths up to 77 MHz^12,79^. In π-FBGs, light is confined to dimensions smaller than those given by the physical length of the gratings. Hence, they feature 1D cavities and a minimum effective sensor size on the order of 10 × 270 µm^80^. As a result, π-FBGs should be treated as line detectors. While they can be used for optoacoustic tomography, they usually provide lower resolution and allow less straightforward image reconstruction than point-like detectors. A nearly ideal sensor geometry can be achieved by creating miniaturized π-BGs in optical waveguides, which can generate greater RI contrast than optical fibers. One example of such waveguides is silicon-on-insulator waveguides^81,82^, for which sensors on the order of 0.5 × 30 µm have been built.

#### Read-out systems

The choice of read-out system for coupling to a sensing mechanism in an interferometric ultrasound detector can strongly condition the performance and potential applications of the detector. For example, the cavity design, acoustic matching, and optical and electrical characteristics of the read-out system govern the sensitivity and bandwidth of resonator-based setups. DI has not been widely implemented for lack of fast, wavelength-sensitive read-out mechanisms. This may change with the recent demonstration of sensitivities down to 8 Pa using confocal Fabry-Pérot interferometers to demodulate wavelength shifts^83^. Although the bandwidth in that system was reported to be only 3 MHz, advances in demodulation methods may increase bandwidth by an order of magnitude.

**Fig. 3:**
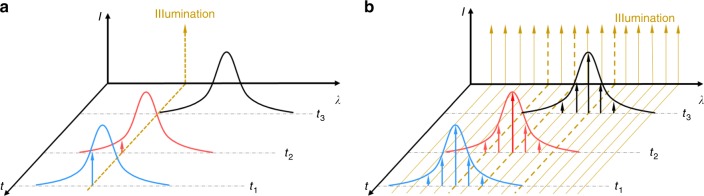
Interrogation methods for optical resonators. **a** In CW interferometry, an interrogation laser is tuned to a wavelength in which the optical spectrum of the interferometer or resonator is approximately linear at time *t*_1_, and the output power is monitored at time *t*_2_. External disturbances and high-amplitude acoustic signals reduce sensitivity and limit the dynamic range at time *t*_3_. **b** In pulse interferometry, the acoustically induced shift of the resonance spectrum is monitored by a broadband pulsed laser. Detecting the spectral shift with an optical demodulator provides high detection sensitivity and dynamic range and allows interrogation of multiple resonators (multiplexing)

Most read-out systems rely on continuous-wave (CW) lasers. In the case of two-beam interferometers or optical resonators, the laser is usually tuned to a wavelength in which the optical spectrum of the interferometer or resonator is approximately linear, and the output power is monitored. The acoustic wave generates stress and strain in the optical sensing element, altering the optical paths within the element and therefore the spectrum, which in turn leads to variations in the monitored power output (Fig. 3a). The fidelity with which this system detects ultrasound depends only on the sensing element, while the noise levels and robustness of the measurements depend on the read-out system. When the sensing element is not acoustically matched to water, acoustic reverberation within the element may lead to signal distortion^84,85^.

CW lasers are also used when sensing ultrasound with DI. Here, the demodulator (Fig. 2c) converts the Doppler shift of the reflected beam to a shift in optical intensity, which is measured by a photodetector. The demodulator is relatively insensitive to low-frequency vibrations because the Doppler shift is proportional to the speed of the vibrating reflectors. In principle, the demodulator can be nearly any optical component whose output changes linearly with wavelength; in practice, confocal Fabry-Pérot interferometers are usually used because of their compactness and high light-gathering efficiency^53,83,86,87^. The linear region of the interferometer resonance is tuned to the laser wavelength such that ultrasound-induced frequency shifts in the laser beam are translated to intensity variations that can be recorded.

Despite its widespread use, CW interferometry has severe disadvantages as a read-out system in optoacoustic imaging. One is difficult scalability: it cannot simultaneously interrogate numerous sensors without scale-up of bulky or expensive components, most notably the interrogating laser itself. Generally, if each sensor operates at a different wavelength, the same number of lasers as sensors is needed to achieve simultaneous read-out. This need for hardware scale-up can be mitigated by tuning all read-out interferometers to the same wavelength as the laser^88^ or by using frequency-modulation schemes that rely on the sinusoidal spectrum of the interferometers^89–91^.

Another disadvantage of CW interferometry is its sensitivity to temperature drifts and vibrations, such as motion of large samples during in vivo imaging. These disturbances, as well as limitations in fabrication accuracy such as local defects in thin-film Fabry-Pèrot interferometers, can shift the resonance spectrum away from the laser wavelength^79,92^, drastically reducing sensitivity and limiting dynamic range (Fig. 3a). This sensitivity to disturbances can be significantly reduced using feedback-based stabilization under carefully controlled conditions^93,94^, but whether this approach works robustly under real-world operating conditions has not been demonstrated. Potentially more robust performance has been obtained using frequency-modulation techniques in two-beam interferometers, such as heterodyne detection^53,57,95^. Pulse interferometry, based on pulsed rather than CW lasers, may offer a more scalable and stable alternative to CW interferometry. In pulse interferometry, optical pulses whose bandwidth is considerably wider than that of the resonator are sent to the resonator (Fig. 3b). The resonator then acts as a spectral filter, such that light exiting it has its spectral shape. An optical demodulator at the output of the resonator detects wavelength shifts. Passive optical demodulators operate stably as long as the spectrum of the resonator is contained within that of the pulses^79^. When the bandwidth of the laser is sufficiently broad, numerous sensors can be interrogated with a single laser by using wavelength-division multiplexing similar to that used in fiber-based sensors of temperature and strain^96^.

## Applications

The advantages of optical over piezoelectric or capacitive ultrasound detection have already been demonstrated in optoacoustic imaging applications; however, they also create the possibility of new optoacoustic applications. In particular, the miniaturization achieved by optical detection of ultrasound could facilitate specialized applications in different fields as summarized in the following.

### Biomedical applications

#### Non-contact detection

An attractive feature of all-optical sound detection in biomedical optoacoustic applications relates to the possibilities enabled by non-contact operation. DI and two-beam interferometry have been used for non-contact optoacoustic imaging^55,88,97–99^ based on optical coherence technology^98,100^ or holographic techniques^101,102^, opening up possibilities for dual-modality imaging. In these configurations, a probe beam is directed onto the sample surface, and vibrations of the tissue edge modulate the reflected light; in other words, the tissue–air interface serves as the reflector (Fig. 2a, c). In this way, for example, a non-contact heterodyne MZI has been used to image blood vasculature in a chicken chorioallantoic membrane in vivo^95^. Non-contact optoacoustic imaging is nevertheless limited by the effects of the large acoustic impedance mismatch at the air-tissue interface^103^ and by inefficient light-collection from rough tissue–air interfaces, leading to low detection sensitivity^56^. This low sensitivity can be compensated to some degree by optically amplifying the initial interrogation beam or the reflected light^83,88,99,104^.

#### Optoacoustic imaging

Piezoelectric transducers typically interfere with light delivery to the sample imaged in optoacoustic tomography setups and necessitate designs that may compromise light delivery. Optical ultrasound detection can be seamlessly integrated with illumination optics because detectors can be made transparent and insensitive to the illuminating light.

**Fig. 4:**
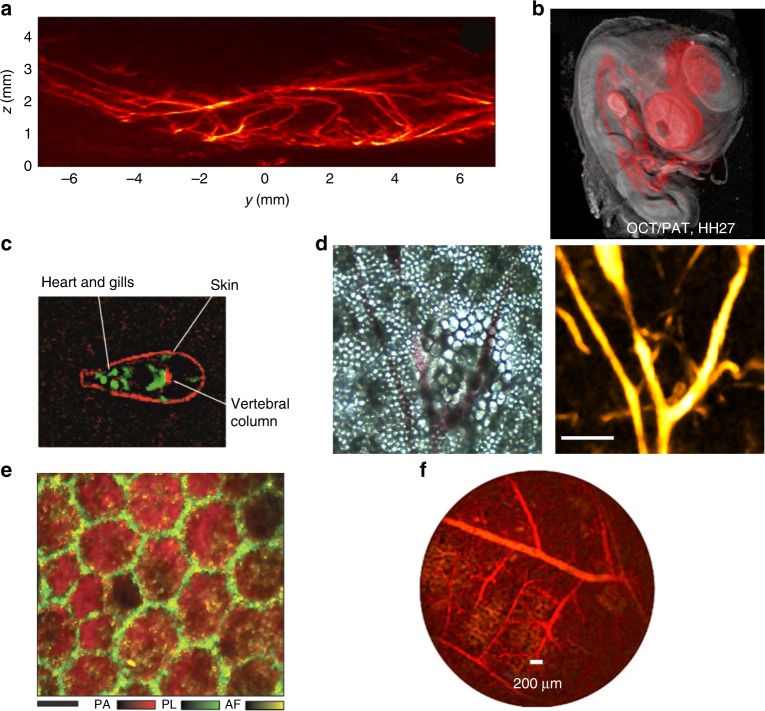
Non-contact optoacoustic imaging based on all-optical sound detection. **a** Optoacoustic tomography image of a hind mouse leg revealing dense microvasculature. Reproduced with permission from ref. ^34^. **b** Optical coherence tomography image of a chick embryo, overlaid with optical absorption contrast (red). Image size, 1.3 × 1.4 cm. Reproduced with permission from ref. ^106^. **c** Zebrafish imaged using pulse-echo, laser-induced ultrasound imaging (red) and optoacoustic tomography (green). Scale bar, 1 mm. Reproduced with permission from ref. ^110^. **d** Bright field (left) and optoacoustic microscopy images (right) of the same region of an ex vivo mouse ear, revealing complementary contrast. Scale bar, 150 µm. Reproduced with permission from ref. ^12^. **e** Human retinal pigment epithelium cells imaged with fluorescence microscopy (green) and optoacoustic microscopy (red). AF autofluorescence, PA photoacoustic maximum amplitude projection, PL phalloidin fluorescence. Scale bar, 10 µm. Reproduced with permission from ref. ^111^. **f** Optoacoustic tomography image of a duck embryo obtained with a setup that can be developed into a fiber-based, forward-looking optoacoustic proof-of-principle endoscope. Reproduced with permission from ref. ^115^

Improvements in the sensitivity of refractometric sensing in general, and phase-sensitive detectors in particular, may support three-dimensional biomedical imaging orders of magnitude faster than existing optoacoustic tomography methods^34^, as recently demonstrated in a study of vasculature in mouse leg (Fig. 4a). In this regard, Fabry-Pérot interferometers offer particular advantages. They are transparent, which means they can be placed in contact with the sample, limiting acoustic attenuation and providing a nearly complete tomographic view of the first few millimeters of tissue. Their wide detection bandwidth, resonance-free acoustic detection spectrum, and small detector area can provide highly detailed images with almost isotropic resolution of 100 µm, which has yet to be achieved in three-dimensional piezo-based optoacoustic systems^1^. Optoacoustic systems based on planar optical resonators have been used for imaging mouse^105^ and chicken embryos^106^ (Fig. 4b), as well as tumors^107^ and brains^76^ of adult mice. Fabry-Pérot-based optoacoustic tomography is limited to imaging at depths of several millimeters, so it has been used clinically so far only for dermal and subcutaneous imaging.

The fact that optical resonators are optically transparent and can be seamlessly integrated with other fiber-based optical imaging modalities makes them well-suited for generating hybrid imaging systems. For example, a planar Fabry-Pérot interferometer has been integrated with a hybrid system combining optoacoustic tomography and optical coherence tomography^106,108,109^, and this system has been used to image mammalian tissue as deep as 10 mm. In a different approach, an MZI has been used to build an all-optical hybrid system combining optoacoustic tomography and laser-ultrasound tomography for imaging zebrafish (Fig. 4c)^110^.

A major challenge for optical ultrasound sensors in optoacoustic tomography remains the parallelized read-out of multiple sensors without duplicating the read-out system. Solutions to this problem may be possible using fiber-based MZIs^59,89^. Moreover, the broad bandwidth achieved by optical sound detection makes optical sound detectors a ubiquitous technology for different implementations of acoustic-resolution optoacoustic imaging, spanning from optoacoustic microscopy to optoacoustic macroscopy applications^1^. Therefore, the same detector could be employed for imaging at different scales, possibly extending the operating characteristics of an optoacoustic system from acoustic resolution microscopy to mesoscopy and macroscopy.

#### Optoacoustic microscopy

Optical resolution optoacoustic microscopy utilizes focused light beams for tissue illumination. The imaging resolution achieved in this case obeys the laws of optical diffraction, not ultrasonic diffraction. Similar to limitations seen in acoustic-resolution optoacoustic imaging, the use of microscope objectives directs the placement of ultrasound transducers away from and possibly at an angle to the volume illuminated. This geometrical arrangement establishes long ultrasound propagation paths, typically through a coupling medium such as water, reducing the effective sensitivity and bandwidth of the setup. In practice, this limits many optoacoustic microscopy setups to transmission-mode geometries, which cannot be used with thicker samples such as animals^3^.

Recently, these limitations have been bypassed using π-Bragg grating based detectors, which have been used to integrate optical-resolution optoacoustic microscopy with transmission-mode bright-field microscopy (Fig. 4d)^12^ and epi-illumination second harmonic generation microscopy^75^. MRRs have been used to integrate confocal and fluorescence microscopy^111^ (Fig. 4e). The broad detection bandwidth of MRRs can provide higher axial sampling resolution than piezo-based alternatives in optoacoustic microscopy^112,113^. For example, an MRR-based system has achieved axial acoustic resolution of ~2 µm^111^, much better than the 15 µm typical of conventional optical-resolution optoacoustic microscopes with piezo-electric detectors^114^.

#### Miniaturization and interventional imaging

Interferometric ultrasound detection systems can be miniaturized to a much greater extent than their piezoelectric counterparts. Sub-millimeter resonator-based detectors have greater sensitivity than similarly sized piezoelectric detectors (Table 1)^12,79^. The fact that optical resonators can be miniaturized and fabricated in optical fibers or coupled to optical fibers makes them highly attractive for minimally invasive optoacoustic imaging in medical endoscopy. Indeed, miniaturized optoacoustic endoscopes have been constructed using Fabry-Pérot interferometers^115–117^(Fig. 4f), MRRs^118^ and π-Bragg gratings^79^, which offer sensitivities as high^92^ as $25\frac{{\rm{mPa}}}{{\surd {\rm{Hz}}}}$. The ultra-small form factors of optical-resonator-based devices may support the development of intravascular ultrasound sensors for analyzing atherosclerotic plaques or implanting coronary stents; intravascular imaging requires catheter sheets with 3-mm diameters (9 F) or smaller. Minimally invasive monitoring of cardiac tissue in vivo has already been achieved in laser ultrasonics involving an optical-fiber-based Fabry-Pérot interferometer for ultrasound detection^119^. The same approach may prove effective for optical detection of sound in optoacoustics, given the parallels between this field and laser ultrasonics.

### Beyond biomedical optoacoustics

All-optical sound detection has been explored in magnetoacoustics and non-destructive testing, where systems are typically exposed to high levels of electromagnetic interference. This noise poses a problem when ultrasound is detected using piezoelectric transducers because the piezoelectric element itself or the signal cables act as antennas. Optical components to detect ultrasound, in contrast, are immune to such electromagnetic interference.

#### Magnetoacoustics

**Fig. 5:**
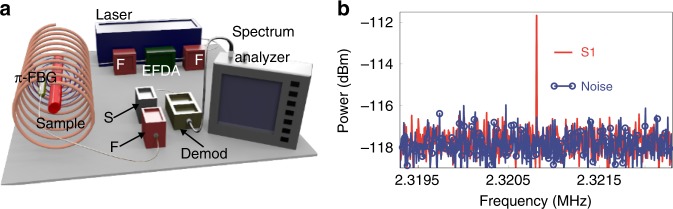
Magnetoacoustic sensing of magnetic nanoparticles. **a** Experimental setup in which a π-FBG-based acoustic sensor is inserted into a magnetic induction coil together with the sample. Demod demodulator, EFDA erbium-doped fiber amplifier, F optical filters, S optical splitter. **b** Acoustic spectra of Fe_3_O_4_ nanoparticles measured at an excitation frequency of 1.1604 MHz as a result of acoustic coupling between the sample and the sensor. The frequency-doubled signal S1 (red) differs from the recorded noise (blue), confirming that the signal is not due to electromagnetic interference. Reproduced from ref. ^120^

Magnetoacoustic imaging devices visualize features of a sample exposed to transient magnetic fields, which trigger the production of ultrasound waves that travel from the sample to a detector^120^. The ultrasound is generated via one of two mechanisms. In the first, a changing magnetic field induces eddy currents that generate internal pressure due to Lorentz forces; the sample can then be imaged based on electrical conductivity^121,122^. In the second mechanism, magnetic energy deposition in the imaged object leads to thermal expansion such as in ultrasound generation in optoacoustics; in this case, the sample is imaged based on magnetic susceptibility^120,123^ (Fig. 5a).

The small magnetic susceptibility of biological samples means that magnetoacoustics cannot generate sufficient contrast for imaging purposes^124^. However, adding magnetic suspensions such as Fe_3_O_4_ nanoparticles to the sample generates contrast based on magnetic susceptibility (Fig. 5b)^120^. Magnetoacoustic measurements can be rendered insensitive to electromagnetic interference using optical detection of ultrasound; this is particularly important when the magnetic excitation is a continuous wave, because time gating cannot be used in this case to reject electromagnetic noise picked up by piezoelectric transducers. Even in the presence of strong contrast due to magnetic nanoparticles, CW magnetoacoustic sensing appears to be practical only with optical detection of ultrasound.

#### Non-destructive testing

Optical methods for ultrasound detection have also been investigated in non-destructive testing, such as for sensing applications in solid materials using two-beam mixing interferometry^55^ or for sensing applications in the related field of laser ultrasonics. First introduced in the 1980s, laser ultrasonics has become a well-established method for the analysis of material properties and detection of structural flaws, mainly in industrial applications^87,125^. The surface of a solid, optically opaque, non-biological sample is illuminated with laser pulses to generate an acoustic wave within a confined area via the optoacoustic effect. The propagation and reflection of this acoustic wave is subsequently monitored using non-contact optical interferometry. Acoustic waves are reflected at defects and flaws in the sample because these regions feature areas of acoustic impedance mismatches resembling metal–air interfaces. Most laser ultrasonic systems use an interferometric setup in which Doppler shifts in frequency are detected^53^. These measurements can serve not only to detect faults but additionally to characterize the mechanical and geometric properties of the sample. An additional approach to detect ultrasound signals in laser ultrasonics is based on beam deflectometry; in this technique, the probe beam is deflected off the surface of the sample and the angular modulation of the deflected beam is recorded using knife-edge or other methods^125^.

Optical detection of ultrasound is critical for laser ultrasonics because it allows non-contact measurement without the need for a coupling agent and because it can operate at high temperatures that piezoelectric transducers cannot withstand. Laser ultrasonics has become a standard tool for inspection of aircraft composites^126,127^, wind turbine blades^128^, nuclear reactor components^129^, and pipe thickness during high-temperature manufacturing^130^.

**Fig. 6:**
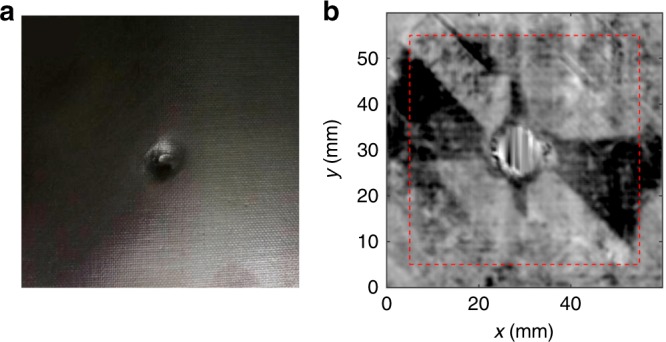
Non-destructive testing of a carbon-fiber-reinforced polymer. **a** Top-view photograph of a 32-layer, fiber-reinforced polymer structure with a defect at its center resulting from a standardized 50-J impact (ASTM D7136). Sample size, 65 × 65 mm. **b** Laser ultrasonic image of the same area of the reinforced polymer reveals propeller-shaped damage below the sample surface. Reproduced with permission from ref. ^132^

Parallel detection schemes have yet to be developed for laser ultrasonics, which means that three-dimensional imaging requires time-consuming scanning. This is not a major concern for industrial samples because acoustic impedance usually varies substantially, so scanning can be accelerated without losing much sensitivity. For example, excitation with lasers at kHz repetition rates can generate three-dimensional images of carbon-fiber-reinforced polymers showing contrast complementary to that of X-ray tomography^131,132^ (Fig. 6).

Laser ultrasonics also shows potential for optoacoustic imaging of biological samples and has been used for microscopy of single cells^133^ and optoacoustic tomography of calf brain^99^. The acoustic signals from biological samples are much weaker than those from industrial samples because acoustic impedance varies less within tissues. It will be interesting to see whether future advances in optoacoustics hardware and image-processing techniques can lead to the mainstream integration of laser ultrasonics into optoacoustic imaging.

## Conclusion

Optoacoustic imaging presents challenges for ultrasound detection different from those found in ultrasonography and has intensified interest in optical ultrasound detection methods. Despite recent research advances, common piezoelectric transducers are opaque and interfere with the illumination requirements of optoacoustic setups. Moreover, it is challenging to miniaturize piezoelectric transducers while retaining broadband detection of several tens of MHz or wider. Optical sound detection can bypass these limitations, setting new performance standards for all-optical optoacoustic imaging. Nevertheless, piezoelectric transducers are generally more economical than optical technologies, especially for large-scale manufacturing. Additionally, piezoelectric sound detection can be achieved without the cost-intensive read-out systems required for optical detectors. Finally, PMUTs and CMUTs can allow manufacturing of high-density detector arrays, which is challenging for optical detectors^81^.

Advances in parallelized all-optical detection have been recently demonstrated, in particular in the fabrication and simultaneous interrogation of interferometric detector arrays^89,91,134–136^. However, piezoelectric detector arrays remain the key technology for real-time optoacoustic imaging in most applications. Therefore, different optical sound-detection technologies may eventually enable different operational regimes depending on the particular specifications of the intended application.

Recent advances in refractometric methods may enable implementations of non-contact optoacoustic imaging. These methods, particularly those based on Schlieren-beam interrogation, may allow ultra-fast optoacoustic tomography that does not require image reconstruction. At the same time, interferometric methods may show greater performance potential in a range of optoacoustic imaging contexts. These methods have already facilitated the development of substantially smaller detectors and have catalyzed improvements in detector sensitivity and bandwidth for biomedical optoacoustic applications^75,79,137–140^. In particular, optical resonators can be miniaturized without compromising sensitivity, opening up new possibilities for optoacoustic endoscopy probes for minimally invasive applications. For example, fiber-based optical resonators have been integrated into hybrid probes that combine all-optical optoacoustic imaging with optical coherence tomography and fluorescence imaging.

Further advances in interferometric and refractometric sensing of ultrasound are likely to bring these methods closer to integration into optoacoustic imaging systems for biological and clinical use. In the meantime, optical detection of ultrasound has already become a viable, even preferred, alternative to piezoelectric detection in several optoacoustic applications.
